# Salmagundi

**Published:** 1886-11

**Authors:** 


### SALMAGUNDI.

EDITED BY E. G. BETTY, D.D.S.

THE patent medicine trade of the United States amounts to $22,000,000 annually.

Antipyrine, according to the German Pharmacopeia Commission, under certain circumstances rivals quinine.

OUR friend the "Italics of Interest" man had a serious relapse in October; there are hopes of his recovery however.

"BRIDGE-WORK" is becoming so much the rage, that dentists will shortly feel themselves competent enough to act as County Commissioners.

HYDROCHLORATE OF CAFFEINE has been observed by Dr. Terrier to possess an anæsthetic action almost identical with that of cocaine.—*Jour. de Med. de Paris.*

A TEASPOONFUL of equal parts of castor oil, glycerine, and sherry, shaken together, provides an efficient purgative in diseases of children.—*Medical World.*

DR A. M. CALLAHAM, of Topeka, Kansas, made a flying visit to Cincinnati last month. He is well and reports the dental world in a flourishing condition in his part of the great West.

THE above is a pleasant method of administering the oil and will frequently give relief to the little patient suffering from the constitutional or reflex effects of retarded or in difficult dentition.

A couple of mastodon teeth were recently unearthed near Dayton, O.; the smaller of the two weighed five pounds and nine ounces and was in an excellent state of preservation.

---

" How doth dose little business bee
Delight to bark und bite,
Und gadther beeswax all dose day,
Und eat him up at night."

---

Sozolic Acid.—This substance, called also orthoxy-phenyl-sulphurous acid, is said to possess superior antiseptic properties. It has been used internally as well as externally in erysipelas, small-pox, pneumonia, phthisis, and other affections, with, it is claimed, excellent results.—*Medical and Surgical Reporter.*

---

Professors Fischer and Penzoldt, of Erlangen, have established the curious fact that the sense of smell is by far the most delicate of the senses. They find that the olfactory nerve is able to detect the presence of 1----2,760,000,000 of a grain of mercaptan.—*Public Opinion.*

---

Coleridge is credited with saying "In the treatment of nervous diseases, he is the best physician who is the most ingenious inspirer of hope." How equally true it is that the best dentist is he who calms the fears and nervousness of a timid patient, and so is enabled to complete a perfect operation, rather than he who gives way to anger and fails to accomplish the work.

---

A novel way of reassuring a patient was lately resorted to by a dentist in Mexico. A patient was to have a tooth extracted, but the preparations so frightened him that he declined to submit to the operation. To allay his fears, the dentist made his office boy open his mouth, and in a twinkling took out one of his teeth. We are not surprised to learn that the little fellow threw up his situation at once.—*Medical and Surgical Reporter.*

Origin of Alcohol.—According to the Talmud, one theory concerning the origin alcohol is as follows, viz.:

Noah planted the first vineyard, Satan being present and assisting in the work. After the vineyard was planted Satan slew a lamb, a lion, an ape, and a hog, and with their mingled blood watered the roots of the vines. This he did, he said, because he who shall taste for the first time of the juice of the grape will be a lamb; he who shall use it in moderation will become—for the time being—a lion; he who shall use it to excess will become an ape, and he whom it shall master will become a hog.—*Medical and Surgical Reporter.*

---

The Air of the Sea.—The air of the sea, taken at a great distance from land, or even on the shore and in ports when the wind blows from the open, is in an almost perfect state of purity. Near continents the land winds drive before them an atmosphere always impure, but at one hundred kilometres from the coasts this impurity has disappeared. The sea rapidly purifies the pestilential atmosphere of continents; hence every expanse of water of a certain breadth becomes an absolute obstacle to the propagation of epidemics. Marine atmospheres driven upon land purify sensibly the air of the regions which they traverse; this purification can be recognized as far as Paris. The sea is the tomb of moulds and of aerial schizophytes.—*MM. Moreau and Miquel.*

---

Commonplace Book.—One of the most useful habits possible to be cultivated by medical men, is that of keeping a commonplace book; in which to record events and notes of practical professional interest. In this way many who pursue such a plan, have found it of inestimable service subsequently to be able, in a moment of emergency to refer for exact information, concerning, *e. g.*, a recipe or mode of treatment to a convenient source, where, at the time of first making it, the observation has been recorded. But few people, however, possess the faculty of arranging the material thus acquired in a way to render it immediately accessible. Its mere inscription in a note book, without some accurate method of classification is adopted, creates coufusion under circumstances in which confusion is least of all desirable; and hence it is, that a reference MS. book has become an important desideratum to every practitioner, who desires to derive permanent advantage to himself and his patients, from the results of his reading and practice.—*Medical Library.*

---

In the latter part of September, we extracted for a woman of about 28 years of age, a supernumerary tooth occupying part of the space left after the extraction some years ago, of the second superior right molar. The first and third molars are in situ. The supernumerary is shaped very much like a bicuspid, though smaller. A few days before this case was seen, another presented itself, though of a different order. A woman of about the same age, both nearly if not quite six feet in height, of fine physique and rubust health (from Indiana) consulted the writer in regard to some fillings. The anomaly consisted in a pair of well shaped bicuspids in place of the superior lateral incisors. Their position in the arch is in regular line with the other teeth, one cusp on the labial aspect, the other and smaller, on the lingual. The fissure and pits in the crown were that of an apparently normal bicuspid. We wished to take an impression of the mouth, but the lady would not consent.

Such instances as these are not phenomenal, though sufficiently rare to be remarkable and worthy of record. We shall be glad to have our readers report to us any such cases as may happen to come under their observation.

